# An Economic, Modular, and Portable Skin Viscoelasticity Measurement Device for In Situ Longitudinal Studies

**DOI:** 10.3390/molecules24050907

**Published:** 2019-03-05

**Authors:** Seungman Park, Jiaxiang Tao, Li Sun, Chen-Ming Fan, Yun Chen

**Affiliations:** 1 Department of Mechanical Engineering, Johns Hopkins University, Baltimore, MD 21218, USA; spark161@jhu.edu; 2 Department of Embryology, Carnegie Institution for Science, Baltimore, MD 21218, USA; taojiaxiangjit9@gmail.com (J.T.); fan@carnegiescience.edu (C.-M.F.); 3 Department of Bioinformatics, Southern University of Science and Technology, Shenzhen 518055, China; lisun97@foxmail.com

**Keywords:** viscoelastic property, skin tissue, age, indentation-based mechanical analyzer, Prony model

## Abstract

A indentation-based device to measure tissue mechanical property was designed and built using over-the-counter and 3D-printed parts. The device costs less than 100 USD and is capable of measuring samples of various geometry because of its modular design. The device is light-weight, thus portable, for measurements that can be performed at different sites. It was demonstrated that the measurement results obtained using our device are comparable to previous observations. The elastic shear modulus of the human skin was in the range of 2 kPa to 8 kPa, and skin tissues in old mice were stiffer than young mice. Mechanical properties of the skin tissues belonging to the same test subject varied depending on the location of the measurement. In conclusion, because our device is economic, modular, portable, and robust, it is suitable to serve as a standard measurement platform for studying tissue mechanics.

## 1. Introduction

Recent evidence strongly supports the possibility that mechanical properties of tissues, including stiffness, elastic modulus, and shear modulus, significantly contribute to the pace of tumor progression. The mechanical properties of the skin tissue, which occupies the largest surface of the body, have also been implicated in the maintenance of homeostasis, or the progression of diseases. For example, it was recently shown that stability of the tumor suppressor p53 could be modulated by mechanosignaling pathways [1]. In particular, many gain-of-function p53 mutants, which promote tumor progression and are commonly detected in skin cancer patients, are made more stable in stiffer microenvironments by avoiding ubiquitin-mediated protein degradation. Furthermore, it was observed that the stiffer extracellular matrix (ECM) promotes invasiveness of melanoma cells [2,3]. Several common drugs used to treat skin cancer were reported to be suboptimal in cancer cells cultured on stiffer substrates [4].

Mechanical properties of the skin tissues are known to vary with age, hydration, thickness, and Langer’s line [5]. Given that stiffness is associated with cancer development, it is important to understand whether and how the variation in skin mechanics affects tumor progression in a quantitative and mechanistic manner. To assess their effect, the first step is to have a comprehensive survey of skin mechanical properties across demographics. For this purpose, a portable, accurate, and cost-effective device capable of in vivo and in situ measurements is desirable to assess a large sample size for general understanding. In addition, because of recent advances in tissue engineering, it has become a common practice to establish an in vitro skin-mimetic model system to study skin physiology. To engineer tissues in mimicry of the mechanical properties of the real skin, a device capable of measuring functional properties of both engineering tissues and the skin in vivo is ideal. In other words, the device should be modular so that probes are interchangeable to accommodate the specific geometry of the sample to be measured. However, to our best knowledge, no device with all these desired attributes is readily available. 

Currently available in vivo techniques to measure the mechanical properties of the skin include indentation [6], suction [7], torsion [8], wave propagation [9], and extensometer [10]. However, most of the devices are custom-built in an individual laboratory; are costly to build, configure, and calibrate; and demand substantial technical experience to operate. The costs of these systems are at least in the range of 5000–15,000 USD. Moreover, the elastic modulus of the skin varies across two to three orders of magnitude, depending on the device being used [11]. Such large variation severely hinders comparison or verification between studies where similar skin treatments are performed, but different devices are used to measure mechanical properties of the skin after the treatment. An economic and straightforward device that can be easily configured, and thereby commonly used, is beneficial for knowledge convergence in tissue mechanics research.

In anticipation of studying skin mechanics with tissue engineering approaches, we designed a non-invasive, modular, and portable device at a low cost (<100 USD) for the in situ assessment of viscoelastic properties of the skin, as well as those of the fabricated tissues for in vitro studies. Our device is coined “indentation-based mechanical analyzer (IMA)”. Its design takes advantage of the recent maker movement, which incorportes the multitude of inexpensive mechanical and electronic parts that are commercially available for consumers. IMA implements an indenter to make prescribed, small deformations in the sample with a linear actuator, and records the reaction force from the indented tissue over time using a force sensor. The actuator and the sensor are controlled by Arduino, a popular electronic board for input/output signals. Because the probe, which consists of the indenter and sensor, is designed to be interchangeable, IMA can measure samples of peculiar geometries by switching between probes of different sizes and shapes. Because IMA is inexpensive, easy to build, and simple to calibrate and operate, we envision that it can be widely adopted by the community of tissue mechanics, and that the values of mechanical properties measured in different laboratories can be directly compared using IMA as a common measurement platform.

As proof-of-concept, we demonstrated that IMA was capable of measuring abdominal skin tissues in a young (3 months) and an old (2.2 years) mice, as well as skin tissues at different sites of human arms. Our test results in mice agree with the previous observation that stiffness increased with age. In addition, we also extracted the parameter associated with skin viscosity, including the stress relaxation time based on the Prony model. The stress relaxation time has been commonly used as the parameter to characterize biomaterials, and has been shown to be associated with many parameters concerning cellular behaviors, including proliferation rates, cell migration speeds, and morphology, among others [12,13,14]. The goodness of fit of our data to the well-tested theoretical model suggested that IMA is suitable for measurement of viscoelastic biomaterials.

## 2. Design of the Indentation-Based Mechanical Analyzer (IMA)

**Figure 1:**
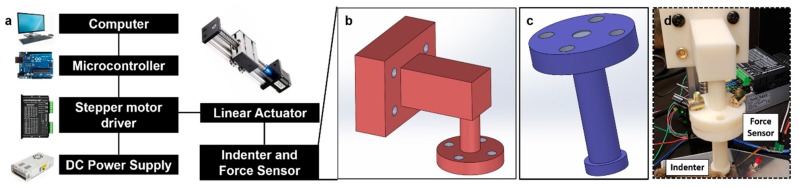
Design of the indentation-based mechanical analyzer (IMA). (**a**). The linear actuator is controlled by a programmed microcontroller which drives the stepper motor. The upper part (**b**) of the indenter is connected to the linear actuator, and its lower part (**c**) is equipped with a force sensor. The flat indenter can be displaced vertically to deform the tissue and record the reaction force from the tissue over time. (**d**). The actual device. The support (white) of IMA is 3D printed and assembled by screws.

IMA was designed to evaluate the viscoelastic properties of the skin by measuring the reaction force originating from the skin tissue upon indentation (Figure 1). A force sensor (15 mm diameter force sensor, SingleTact, CA, USA)and a linear actuator, coupled with a blunt-ended indenter, were integrated to permit real-time measurements (Figure 1a). The linear actuator consisted of a SFU1605 linear guide rail with a 23Nema stepper motor purchased from amazon.com. To temporarily deform the tissue with a dent of prescribed length, the indenter was displaced by the linear actuator, which was connected and controlled by the stepper motor driver. The microcontroller on the Arduino board connected to the computer sent the electric signal to the stepper motor driver, based on user inputs. The cylindrical indenter was fabricated by a high-resolution 3D printer (ABS-M30™, Stratasys Ltd., Eden Prairie, MN, USA) (Figure 1b,c) and coupled with the linear actuator using screws. The stl files for cylindrical indenters of various geometries are available (Supplemental File 1). The force sensor was attached to the front end of the circular indenter to measure the reaction force with which the indenter is in contact (Figure 1d). 

It should be noted that during the measurement, the probe of IMA, though only in contact with the epidermis, results in measurements that reflect the integrated mechanical properties of tissues underneath the epidermal layer, including dermis, hypodermis, adipose tissue, and skeletal muscles. Such inclusion is inevitable in in situ indentation methods [15], not limited only to IMA. However, it is not disadvantageous to obtain the integrated mechanical property as the measurement. In fact, it is more informative and physiologically relevant to measure the integrated mechanical property of skin, adipose, and muscle tissues, since cells in general sense the stiffness of the substrate at a depth of multiple cell body lengths and respond accordingly [16,17]. Moreover, it has been recently confirmed that skin, adipose, and muscle tissues crosstalk and regulate the homeostasis of each other through a complex network that involves mechanosignaling [18,19,20,21,22]. Therefore, the overall mechanical properties of the tissue environment, which include tissues underneath the skin, should be taken into account when studying the effect of mechanical cues originating from the environment on skin health. 

## 3. Results

### 3.1. Characterization of Viscoelastic Properties of Abdominal Skin in Mice

**Figure 2:**
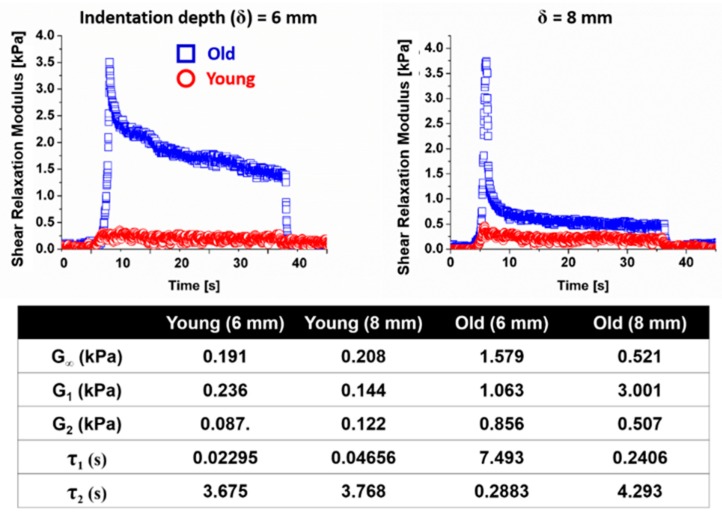
Measurement of the shear modulus and relaxation time of the abdominal skin in mice. The shear moduli and relaxation times of the abdominal skin were obtained from the IMA measurement. Two indentation depths, 6 mm and 8 mm, were implemented. It was observed that the older mouse was stiffer and more viscous with longer relaxation time.

Using IMA, viscoelastic properties of a 3-month-old young female mouse and 2.2-year-old female mouse were measured. The shear moduli, expressed as the sum of G_1_ + G_2_ + G*_∞_*, under 6 mm and 8 mm indentation depths with respect to time were 0.514 kPa and 0.474 kPa in the younger mouse and 3.498 kPa and 4.029 kPa in the older mouse, respectively (Figure 2). It was observed that the older mouse exhibited higher stiffness, expressed as the sum of G_1_ + G_2_ + G*_∞_*, regardless of the indentation depth (Figure 2). In addition, the overall relaxation time (τ_1_ + τ_2_) of the older mouse was two-fold longer than that of the younger mouse.

### 3.2. Characterization of Skin Viscoelastic Properties of the Human Arm

**Figure 3:**
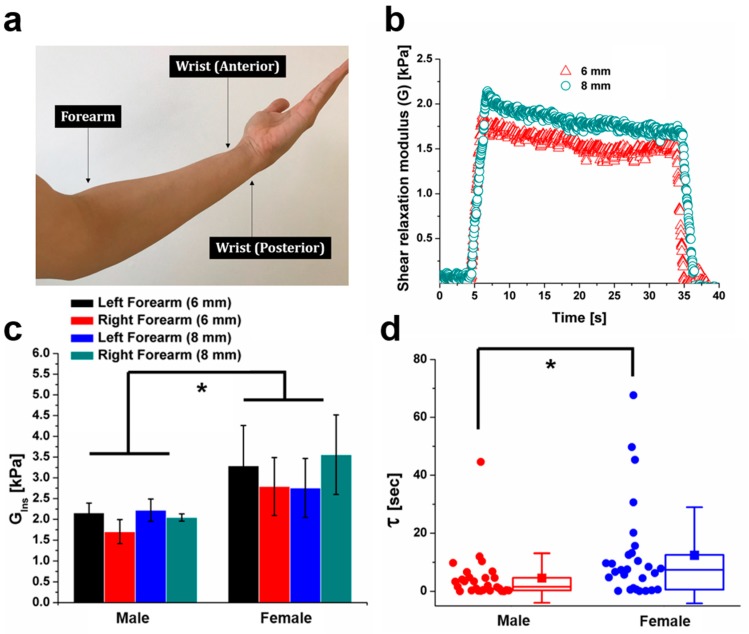
Measurement of the viscoelasticity of the human skin tissue. (**a**) Three different locations of the skin tissues in the arm were measured: forearm, anterior wrist, and posterior wrist. The measurement was performed at the left and right arms of a female volunteer and a male volunteer who were 30–40 years old. (**b**) Representative shear relaxation modulus curve for right forearm of the female. (**c**,**d**) Instantaneous shear modulus (*G_inst_* = *G*_0_ + *G*_1_ + *G*_∞_) (**c**) and relaxation time (τ) distribution (**d**) under 6 mm and 8 mm indentation depths, obtained based on the measurements. (**c**) Data are presented as means ± standard deviation (*n* = 4). (**d**) Box and Whiskers are mean and standard deviation respectively, and box represents the 75th percentile, median, and 25th percentile (*n* = 28). * *p* < 0.05.

The stress relaxation responses of three different parts of the skin tissue at the lower arm—forearm, anterior wrist, and posterior wrist (Figure 3a)—were examined using IMA. A male and female human subject between 30 and 40 years old were tested. The skin at the forearm exhibited a typical viscoelastic behavior (Figure 3b). Overall, the measured instantaneous shear modulus for the male and female subjects was shown to range from 1.70 kPa to 3.56 kPa (Figure 3c). It should be noted that both instantaneous shear moduli and relaxation times of the female skin were, in general, higher than those of the male skin, which agreed with previous observation [23,24,25] (Figure 3c,d). The average relaxation time of the female subject was 12.4 sec, which was about 2.7 times higher than the male subject with 4.6 s.

**Figure 4:**
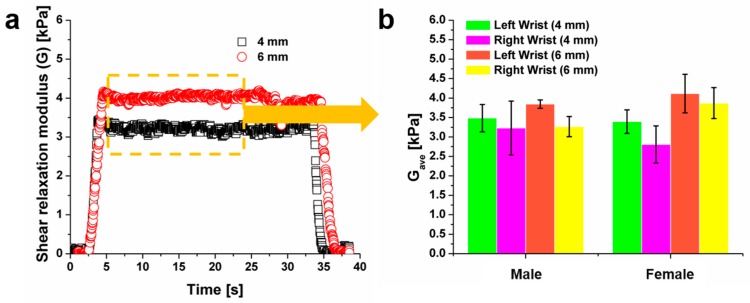
Comparison of the average shear modulus of the left and right anterior wrist using 4 mm and 6 mm indentation depths from the male and female subject. (**a**) The shear relaxation modulus was shown to be similar during the stress-relaxation test, indicating an elastic behavior. (**b**) The elastic shear moduli in orange dashed line (**a**) were averaged to yield average shear modulus (G_ave_).

In contrast to the viscoelastic responses observed in the skin tissue at the forearm, the skin tissues at the anterior and posterior wrist areas exhibited responses dominated by elasticity to the indentation (Figure 4a). We observed that the elastic moduli of skin at the anterior wrist tissues of female and male were similar, ranging from 3–4 kPa (Figure 4b).

**Figure 5:**
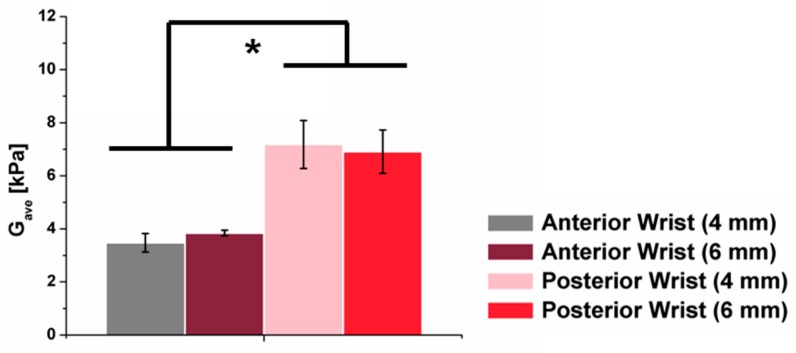
Comparison of the shear modulus of the left anterior and posterior wrists using 4 mm and 6 mm indentation depths from the male test subject.

Our results revealed that the skin at the anterior wrist was stiffer than the forearm by 1.5–2 fold (Figure 3c and Figure 4b), which agreed with the anatomic structure of the human body, where the soft adipose tissue was thicker at the forearm. By the same token, given that the soft adipose tissue was slightly thinner at the posterior side of the wrist, the elastic shear moduli of the skin at the posterior wrist was 2 times higher than those of the anterior wrist, irrespective of indentation depth (Figure 5). Based on this measurement, it can be summarized that forearm tissues showed a viscoelastic behavior, whereas anterior and posterior wrist tissues showed an elastic behavior. In addition, the mechanical properties varied depending on the part of the tissues. Posterior tissues were stiffest, followed by anterior tissues and the forearm.

## 4. Discussion

### 4.1. IMA is an Economic, Versatile, and Robust Device in Measuring Mechanical Properties

In this study we developed a device that was assembled from widely available electronic components and 3D-printed parts, under the budget of 100 USD, to measure viscoelastic properties of tissues in situ. Through calibration by known weights, we first verified that the IMA is equipped with adequate force-sensing capacity. By testing the reaction forces of a purely elastic sample made of polydimethylsiloxane (PDMS) with a known elastic modulus, we demonstrated that measurement by IMA qualitatively and quantitatively reflected the mechanical properties of PDMS. The modular design of IMA allowed us to change the probe depending on the geometry of the target location, so that we were able to measure the skin tissue in both mice and human subjects. The lightweight feature of IMA enables mobile measurements, which will be beneficial when a large sample population of human subjects is to be measured. Moreover, because of its simple design and assembly, in addition to low cost, we envision that IMA can be widely adopted by the biomechanics community as a common measurement platform so that the variation in measured values of biological samples will not originate from the choice of measurement methods. 

### 4.2. The Measured Values of Mechanical Properties Match with the Previous Findings

The measurement results by IMA on human skin are comparable to some of the previous studies (Table 1). For example, Boyer et al. reported that the elastic modulus of skin at the human forearm ranged from 5 to 15 kPa using an air flow system [26]. Elastic moduli of the human skin evaluated by dynamic indentation were reported to be 7–22 kPa [27,28,29]. In summary, we observed an agreement between our results and previous studies in terms of the order of magnitude, supporting the notion that IMA and the accompanying data analyses are suitable for in situ tissue measurements. 

### 4.3. The Variation of Mechanical Properties Observed Using IMA Agrees with Previous Studies

The measurement results of the skin tissues for the young and old mice at the abdominal area showed that the tissue of the skin of the old mouse was stiffer with a longer relaxation time, compared to the young mouse. Our observation agreed with the notion that aged skin tissues [5,30,31], as well as other collagen-rich tissues such as corneal [32] and bone [33], are stiffer as reported previously. A widely accepted hypothesis is that the increased stiffness in skin during the aging process stems from the reduced water content [34]. Alternatively, it has been proposed that the abundance of chondroitin-sulfate and keratin-sulfate in the dermis declines with age, resulting in collagen fibers that are more resistant to elastic deformation [5]. It is also possible that the total collagen content in the skin tissue [35], as well as other collagen-rich tissues underneath the skin [36], decreases during the aging process, subsequently leading to the increased elastic modulus. We observed that the skin tissue in the old mouse exhibited a longer relaxation time (Figure 2), a parameter of the viscosity. To the best of our knowledge, currently, there has been no study investigating the relationship between age and the viscosity parameter of the skin, though it has been implied that tissue viscosity plays an important role in homeostasis [13]. Though our small sample size limits the conclusiveness of our results, future IMA use can serve as the measurement platform for skin viscosity studies where adequately large sample sizes are surveyed. 

Using IMA, we observed that the skin tissues at different body locations of the human subjects vary in their viscoelastic properties, agreeing with previous studies by tensile strength measurement [37], optical coherence tomography (OCT) [38], and suction-based measurement [39]. In the future, it will be interesting to explore whether the variation in skin viscoelastic properties correlates with the variation of the incidence of skin diseases by implementing IMA.

### 4.4. Limitations and Perspectives

In this study, by testing the same subjects multiple times, we confirmed the measurement reproducibility of IMA. Based on the reproducible results, we showed that IMA is feasible in measuring the mechanical properties *in situ*, Moreover, the agreement between IMA measurements and other studies on the PDMS block should establish the validity of IMA. Indeed, the design principle of IMA is based on dynamic measurement of the response to indentation in biomaterials, which has been long established. In this study we leveraged the advances in electronics and 3D printing to develop an affordable and open-sourced version of the device. The Prony series, which was used to extract the stiffness and relaxation times in our study, is also well-established and commonly used. Therefore, the measurement results obtained by IMA can be trusted in terms of validity. While the absolute values of skin stiffness and relaxation time might not be identical to previous measurements, likely resulting from instrumental variations when different types of devices were used, we made the same qualitative observations in the skin tissues: old skin tissues are stiffer, and skin viscoelasticity varies depending on the site of measurement. It should be noted that the IMA measurement results in this work, while comparable to previously reported observations, were obtained from very small sample sizes and only for the purpose of demonstrating the feasibility of in situ tissue measurement using IMA; therefore, the results not suitable for scientific interpretation. In the future, studies involving statistically significant sample sizes should be carried out to confirm our observation using IMA. 

## 5. Materials and Methods

### 5.1. Experimental Procedures

Stress relaxation measurements were performed using IMA to measure the mechanical properties of the tissue environment in which skin cells sense. In this study we used 6 mm and 8 mm indentation depths for the mouse belly and human forearm, and 4 mm and 6 mm for the human anterior and posterior wrist, respectively. During the measurement, a prescribed constant displacement was applied to the tested tissue, and the magnitude of the resulting reaction force was recorded over time. The Arduino codes that control the indenter displacement is available in the Supplemental Materials (Supplemental File 2). The elastic and shear moduli, and the characteristic relaxation time of the skin tissue, were calculated based on the recorded force data over time. The calculation was performed by fitting the experimental data to the Prony model. 

### 5.2. Force Calibration and Validation of Measured Properties

**Figure 6:**
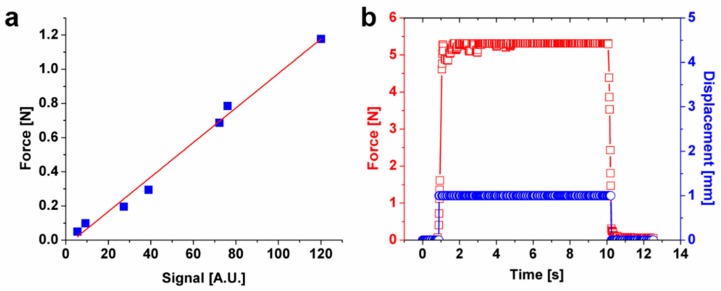
Force calibration and validation test of mechanical properties using polydimethylsiloxane (PDMS). (**a**) The force sensor was calibrated using different scale weights. (**b**) A stress relaxation test was performed using PDMS of known mechanical properties. A constant displacement (1 mm) was applied to the PDMS, and the reaction force was measured over time. The measured curve shows the typical elastic behavior.

A force calibration test of IMA using standard weights was performed (Figure 6a). The load–force curve showed that the signal generated by the force sensor was linearly proportional to the magnitude of imposed force from the standard weights. Next, we tested IMA by measuring a substrate with known mechanical properties. A polydimethylsiloxane (PDMS) block of 5 mm thickness was fabricated by mixing part A and part B of Dow SYLGARD 184 (Corning) at a ratio of 10:1. The PDMS block was then indented by 1 mm, and the reaction force was profiled using IMA (Figure 6b). The reaction force stayed constant during the measurement, exhibiting a typical elastic behavior. Based on Hooke’s law, the elastic modulus of PDMS was determined to be ~100 kPa, comparable to values previously reported (~10 kPa–10 MPa) [40].

### 5.3. Stress Relaxation Test Using Mouse and Human Models

**Figure 7:**
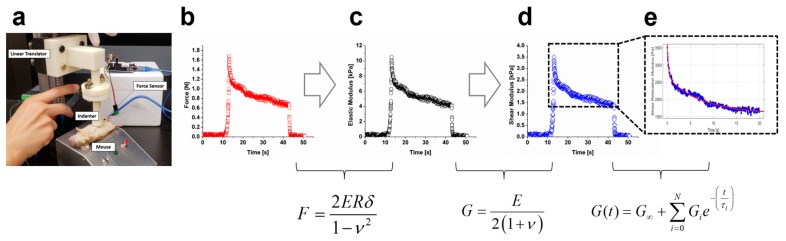
In vivo measurement of the viscoelasticity of the abdominal skin in mice. (**a**) During the measurement, a mouse is placed supinely on the platform underneath the indenter. (**b**–**e**) The prescribed displacement of the indenter compressed the skin tissue, from which the passive reaction forces can be measured over time (**b**). The value of the reaction force measured immediately upon the indentation was used to compute the dynamic elastic modulus (**c**) and dynamic shear modulus (**d**). The values of the shear modulus over time can be used for fitting the Prony viscoelastic model to extract shear modulus and relaxation time (**e**).

A stress relaxation test was implemented, where the tissue was subjected to constant displacement or strain over the duration of the test, and the force was measured over time. Abdominal skin tissue was tested in mice; three different parts of the arm were tested in human subjects: forearm, anterior wrist, and posterior wrist. For each test site, the measurement was repeated at least three times to ensure reproducibility of the device. The cylindrical indenter (diameter = 20 mm) was positioned such that it barely touched the skin of a test subject (Figure 7a). A constant displacement was applied for 30 s using the programmed linear translator. The sampling rate of the force sensor was set at 33 Hz. The approaching and withdrawing speed of the intender was set at 3.8 mm/second. The reaction force magnitude immediately after the indentation $F_{0}$ was used to estimate the instantaneous elastic modulus using the following relation [41] (Figure 7b,c):

(1) $$F_{0}=\frac{2E_{inst}R\delta }{1-\nu^{2}}$$

where $E_{inst}$ represents the instantaneous elastic modulus, *R* represents the radius of the indenter, δ represents the depth of the indentation, and *v* represents Poisson’s ratio. We adopted the general assumption that *v* was equal to 0.5 for incompressible biomaterials. Assuming that the skin was mechanically isotropic, the instantaneous shear modulus, $G_{inst}$, was calculated using the relation (Figure 7d):

(2) $$G_{inst}=\frac{E_{inst}}{2\left(1+\nu \right)}$$

The time-dependent viscoelastic response can be represented by the Prony series [42], which is expressed in terms of shear relaxation modulus and relaxation time:

(3) $$G\left(t\right)=G_{\infty }+\overset{N}{\underset{i=1}{\sum }}G_{i}e^{-\left(\frac{t}{\tau_{i}}\right)}$$

where *G_∞_* represents steady-state stiffness, *G_i_* represents the shear modulus of the *i*th term, and *τ_i_* represents the relaxation time of the *i*th term. The shear modulus derived from experimental observations of the dynamic force response at each time point was fitted to the Prony model with N = 2 (Figure 7e).

## 6. Conclusions

In this work we presented IMA, a device designed for in situ stress relaxation measurements of tissue, and the accompanying workflow to evaluate the viscoelastic properties of the tissue that was based on Prony model. The proof-of-concept experiments were performed, where measurements by IMA yielded results comparable to data obtained from more expensive and/or complicated devices. IMA is low-cost, straightforward to build, light-weight (thereby portable), and modular (thereby versatile). These properties allow the measurement of samples with various geometries. We envision that IMA will be commonly used by the biomechanics community to study the mechanical properties of tissues in situ as well as tissues engineered in vitro, so that knowledge convergence and advances in tissue mechanics research can be further promoted.

## Figures and Tables

**Table 1:** Representative elastic modulus (also known as Young’s modulus) of human male skin tissues measured in this study.

Location	Elastic Modulus	Displacement
Forearm		
Left	6.5 ± 0.7 kPa	6 mm
	6.7 ± 0.8 kPa	8 mm
Anterior wrist		
Left	10.4 ± 1.1 kPa	4 mm
	11.5 ± 0.3 kPa	6 mm
Posterior wrist		
Left	21.5 ± 2.7 kPa	4 mm
	20.7 ± 2.5 kPa	6 mm

## Supplementary Materials

The following are available online. Supplemental File 1: Indenter designs for 3D printing. Supplemental File 2: Arduino codes to control stepper motor displacement.
